# Allergic diseases of the skin and drug allergies – 2020. The association between DRESS and the diminished numbers of peripheral B lymphocytes and natural killer cells

**DOI:** 10.1186/1939-4551-6-S1-P107

**Published:** 2013-04-23

**Authors:** Mehtap Yazicioglu, Reyhan Elmas, Burhan Turgut, Tugba Genchellac

**Affiliations:** 1 Pediatrics and Pediatric Allergy, Trakya University, Edirne, Turkey; 2 Pediatrics, Trakya University , Edirne, Turkey; 3 Internal Medicine, Trakya University, Edirne, Turkey

## Background

Drug reaction with eosinophilia and systemic symptoms (DRESS) is a drug-induced,severe multiorgan system reaction whose exact pathogenesis remains unknown. This study aimed at evaluating specific changes in peripheral blood lymphocyte subtypes associated with DRESS during antibiotic treatment.

## Methods

We analyzed six patients with DRESS. A complete blood count and peripheral blood lymphocytes immunophenotyping were carried out at symptom onset and at follow-up visits. Acute-phase reactants and liver enzymes were measured in all patients. Other tests – viral serology, serum immunoglobulin levels, and skin tests were performed when possible.

## Results

B-cell counts were low in all patients at the onset of DRESS, and natural killer (NK) cells were low in all cases exceptone. During recovery, B-cell numbers were within a normal range in five patients. In one, there was even a 10-fold increase in B-cell counts, although the level was mildly low after 3 months. NK-cell numbers were within a normal range in three patients. The mean numbers of B cells and NK cells were significantly higher in the second samples compared to the values on admission. Serum IgA and IgM levels were low in one patient. The drug provocation test was positive with cefotaxime in one patient. Viral serology, performed on five patients, was negative.

## Conclusions

A decrease in B-cell and NK-cell counts was the most consistent finding associated with the onset of antibiotic-induced DRESS in our patients. This immunologic alteration might be a useful predictor of DRESS development.

